# Serum Inflammatory Cytokines and Depression in Coronary Artery Disease

**DOI:** 10.5812/ircmj.17111

**Published:** 2014-07-05

**Authors:** Mohammad Tajfard, Latiffah A Latiff, Hamid Reza Rahimi, Mohsen Mouhebati, Habibollah Esmaeily, Ali Taghipour, Elahe Mahdipour, Hafezeh Davari, Zahra Saghiri, Parichehr Hanachi, Majid Ghayour Mobarhan, Gordon A Ferns, Maryam Azizian

**Affiliations:** 1 Department of Community Health, University Putra Malaysia, Kuala Lumpur, Malaysia; 2 Department of Health and Management, School of Health, Mashhad University of Medical Sciences, Mashhad, IR Iran; 3 Health Sciences Research Center, Mashhad University of Medical Sciences, Mashhad, IR Iran; 4 Department of Modern Sciences and Technologies, Mashhad University of Medical Sciences, Mashhad, IR Iran; 5 Department of Cardiology, Ghaem Educational Hospital, Mashhad University of Medical Sciences, Mashhad, IR Iran; 6 Department of Biostatistics, School of Health, Mashhad University of Medical Sciences, Mashhad, IR Iran; 7 Department of Biology-Biochemistry, Payame Noor University of Mashhad, Mashhad, IR Iran; 8 Department of Biology, Biochemistry Unit, Alzahra University, Tehran, IR Iran; 9 Cardiovascular Research Center, Mashhad University of Medical Sciences, Mashhad, IR Iran; 10 Division of Medical Education, Brighton and Sussex Medical School, Brighton, UK; 11 Department of Nutrition, Mashhad University of Medical Sciences, Mashhad, IR Iran

**Keywords:** Coronary Artery Disease, Depression, Cytokines, Tumor Necrosis Factor, Interleukin-8

## Abstract

**Background:**

Severe depression may be accompanied by immune dysregulation and is also associated with increased risk of coronary artery disease (CAD).

**Objectives:**

We investigated serum levels of 10 cytokines and their relationship with depression in patients with cardiovascular diseases as well as healthy subjects in northeast of Iran.

**Patients and Methods:**

The study was carried out on 462 subjects (120 healthy subjects and 342 candidates undergoing angiography). The healthy subjects were referred for routine annual checkups or pre-employment examinations; they did not have clinically evident CAD. A questionnaire was used to obtain demographic data and the Beck depression inventory (BDI) was applied to assess depression. The Evidence Investigator^®^ platform was used for cytokines assays for IL-1α, IL-1β, IL-2, IL-4, IL-6, IL-8, IL-10, TNF-α, MCP-1 and IFN-γ, using sandwich chemiluminescent method. The statistical analysis was performed using SPSS version 11.5.

**Results:**

The mean age was 53.3 ± 11.5, 54.8 ± 11.3, and 59.5 ± 11.3 in healthy, angiography (-), and angiography (+) subjects, respectively (P < 0.05). There were significant differences in serum levels of IL-4, IL-6, IL-10, and MCP-1 cytokines, comparing subjects with CAD and healthy persons (P < 0.05). When all subjects were divided to with and without depression regardless of their cardiovascular status, there was a significant difference in serum levels of IL-8 and IL-6 between the groups (P < 0.05). When the subgroup with features of CAD was selected and divided to those with and without depression, there was also a significant difference in serum levels of IL-8 and TNF-α (P < 0.05).

**Conclusions:**

The positive interaction between depression and CAD was probably mediated by inflammatory mechanisms.

## 1. Background

Severe depression appears to be accompanied by immune dysregulation (1-3). Peripheral cytokines can been influenced by the neuroendocrine system (4), which is modulated by corticosteroids derived from the adrenal cortex (5). Plasma concentrations of proinflammatory cytokines IL-1 and IL-6 increase in patients with depression (6); in patients taking antidepressant medications, reduction of plasma IL-1β appears to be associated with the treatment (7). Development of depression is also associated with elevated circulating concentrations of inflammatory biomarkers; for example, proinflammatory and antiviral cytokines (IL-2, TNF-α and IFN-α) have been associated with flu-like and depressive symptoms (4).

In depressed individuals, there are increased concentrations of several inflammatory biomarkers (8), including TNF-α (9), IL-4 (9), and CRP (10, 11), which can predict cardiac morbidity and mortality (8).

There are associations between inflammation trigger factors and occurrence of coronary artery disease (CAD) (12). In CAD, lipids accumulate in the intima of coronary arteries which is associated with mononuclear cell infiltration and smooth muscle proliferation (13). Traditional cardiovascular risk factors, chronic inflammation, and sedentary life style play important roles in increasing the CAD risk (14). Diet can also significantly affect T-cell mediated immune response, while it appeared to have no effect on B-cell function or production of proinflammatory mediators (15). Cytokines play major roles in activation of adhesion molecules and chemokine expression, involved in leukocyte recruitment (16).

Some investigators have proposed that TNF-α, IL-2 and IL-10 may be potential markers in the prediction of cardiovascular events. C-reactive protein (CRP) and IL-6 have been also identified as independent risk factors for future myocardial infarction (17).

## 2. Objectives

In this study, we investigated serum levels of 10 cytokines and their relationships with presence of CAD among individuals referred for angiography as well as healthy subjects.

## 3. Patients and Methods

The case-control study was carried out on 245 male and 217 female subjects. These included 342 individuals with signs or symptoms of cardiac disease and were candidates for routine angiography according to standard indications. These patients underwent coronary angiography at the Ghaem Medical Educational Hospital, Mashhad, Iran (a tertiary referral center of Khorasan Razavi province). According to the angiography results, the latter individuals could be divided to two subgroups: those with angiographically defined CAD [angiography (+)] (case group) who had more than 50% occlusion in at least one coronary artery and those with a normal angiogram or less than 50% obstruction in coronary arteries [angiography (–)].

Healthy subjects (n = 120) were referred for an annual checkup or pre-employment examination. They did not have clinically evident CAD and were therefore not eligible for angiography; thus, they were assigned as the reference group. In this research, the following formula was used to determine the sample size:

$$n=\frac{{\left(z_{\alpha }+z_{1-\beta }\right)}^{2}(s_{1}^{2}+s_{2}^{2})}{d^{2}}$$

Zα = 0.01 is 2.81, Zβ = 0.10 is 1.28, test power is 90%, S_1_ and S_2_ are standard deviations of groups 1 and 2.

Based on the above formula, a minimum size of 80 samples was determined and 50% (18) was required to cater for nonresponders. The number of samples needed to be recruited was 120 (n = 80 + 40 = 120) samples per group. However, to gain a higher validity and extend the debate to society as a whole, and also because of the probability in sampling, the number of sample in the case group increased as much as three times.

Demographic data and a score for depression were obtained using the following questionnaires:

1. A general questionnaire including participants’ demographic information, with data on anthropometric parameters.
2. Beck depression inventory (BDI) (19, 20).

If the subject’s score was less than 13, he/she was categorized as not depressed, and if the BDI score was more than 13, the subject was categorized as significantly depressed (21).

Persian format of Beck questionnaire was used for depression assessment in this study; its validity and reliability were checked before by some researchers (Cronbach’s α = 0.87 and acceptable test-retest reliability (r) = 0.74) (18, 22-25). All the questioners were filled by one observer. The necessary data had been gathered since September 2011, which lasted nearly more than 20 months.

The inclusion criteria of the healthy group were: being an adult (beyond the age of 18), understanding the study procedures and consent to participate in the study, able and willing to provide written informed consent, having good health condition, no symptoms of heart disease, not being pregnant or in breast-feeding period, no history of hospitalization for any illness during the past five years (26, 27).

Blood pressure was measured using a standard mercury sphygmomanometer. Blood samples (20 mL) were taken in the early morning after an overnight fasting into plain vacutainer tubes and samples for fasting blood sugar measurement were taken using vacutainer tubes containing fluoride-oxalate (28).

Samples were centrifuged to separate cells and serum and serum samples were stored at -80°C until analysis. Total cholesterol, low density lipoprotein, high density lipoprotein, cholesterol, and glucose were measured using standard techniques on a Cobas auto-analyzer system (ABX Diagnostics, Montpellier, France) (28).

According to the American Diabetic Association criteria, a fasting blood sugar (FBS) < 110 mg/dL is considered normal (normal range is between 110 and 126 mg/dL), and FBS > 126 mg/dL is considered as impaired fasting glucose (IFG) and DM, respectively (28).

### 3.1. Laboratory Measurement of Cytokines

An Evidence Investigator® analyzer was used for cytokines assays. The Evidence Investigator can simultaneously detect multiple cytokines from a single sample and uses sandwich chemiluminescent methods. In this study, 10 cytokines (IL-1α, IL-1β, IL-2, IL-4, IL-6, IL-8, IL-10, TNF-α, MCP-1, IFN-γ) were measured simultaneously.

Serums were separated from the blood samples and kept at -80ºC. Analysis of the serum cytokines was performed by an EV 3513 cytokine biochip array (Randox Laboratories, Crumlin, UK), using sandwich and competitive chemiluminescence immunoassays, previously explained by Randox Laboratories (29, 30).

### 3.2. Statistics

The statistical package for social sciences (SPSS version 16) was used for data analysis. The Kolmogorov-Smirnov test was used to assess normality. Descriptive statistics (frequency, mean and standard deviation) were determined for all variables. Values were reported as mean ± SD for normally distributed variables (or median and IQR for not normally distributed variable). Baseline demographics and clinical characteristics were compared among groups using t-student test, one-way ANOVA test, chi-square test, and/or Fisher exact test, as appropriate. Regression modeling analysis was used to remove the effects of confounding factors (such as age, body mass index (BMI), medication (statins), and gender) of CAD and serum cytokine concentrations. P < 0.05 was regarded as statistically significant.

## 4. Results

The mean age of subjects in the healthy group was 53.3 ± 11.5 years. The mean ages of subjects in the angiogram-negative and angiogram-positive groups were 54.8 ± 11.3 and 59.5 ± 11.3 years, respectively. There was a statistical difference in age between the groups (Table 1). A significant difference was also observed between these groups in gender distribution; 61% of angiogram-positive group were males and 49% and 42.5% of the angiogram-negative and healthy groups were males, respectively (P = 0.003) (Table 1).

More than 60% of subjects who had some signs or symptoms of CAD were overweight or obese; but in the healthy group, 65% had a normal BMI (P = 0.001) (Table 1).

Surprisingly, there was no significant difference in the smoking status among subjects in the three groups (P = 0.297). However, the three groups differ significantly for the presence of diabetes mellitus (P < 0.001). LDL cholesterol was significantly different between healthy subjects and the two other groups (P < 0.05) (Table 1).

Serum IL-4, IL-6, IL-10, and MCP-1 concentrations are shown in Table 1, which were significantly different between the angiogram-positive and healthy subjects (P < 0.05). Considering the depression score and depression category for the three groups, a significant difference was found between the healthy subjects and those with or without angiographically significant CAD (P < 0.05), which is also shown in Table 1.

**Figure 1.**
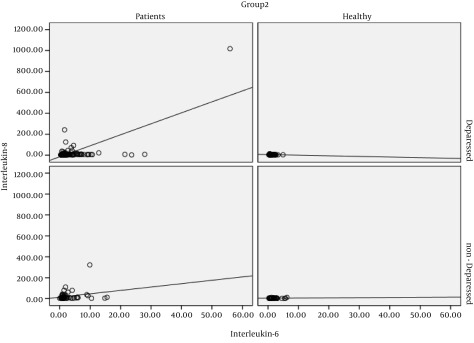
Correlation Between IL and IL8 Serum Levels in Healthy and Patients' Groups According to the Depression Status

Table 2 shows that in depressed and non-depressed groups, regardless of their cardiovascular status, there was a significant difference between IL-8 and IL-6 concentrations. In subjects undergoing angiography, there was also a significant difference in the levels of IL-8 and TNF-α (P < 0.05) between depressed and non-depressed groups. According to Figure 1, one positive linear correlation was found between IL-6 and IL-8 in healthy and patient groups in association with depression status in all subjects.

We adjusted the regression models to investigate the adjusted associations between the groups (healthy, angiography-negative and angiography-positive) as well as the variables mentioned below. The model was used to remove the effects of high BMI (adipose tissue), medications (statins and aspirin), age, and gender, on cytokine and growth factor concentrations. In this model, significant difference was found among the groups in IL-4, IL-6, IL-10, and MCP-1 serum levels [P < 0.05 (Table 3)].

**Table 1.** Characteristics Data From all Subjects and Cytokines and Depression Statues Evaluations in Each Group^a^

Variables	Healthy (n = 120)	Angiography- (n = 111)	Angiography+ (n = 231)	P_0_ (h/a-)	P_1_ (a-/a+)	P_2_ (h/a+)
**Age, y**	53.26 ± 11.53	54.78 ± 11.27	59.47 ± 11.30	0.565	< 0.001	< 0.001
**Sex**				P = 0.003		
Male	51 (42.5)	54 (49)	140 (61)			
Female	69 (57.5)	57 (51)	91 (39)			
**BMI, kg/m** ^**2**^				P < 0.001		
Normal	78 (65)	38 (35)	87 (42)			
Overweight	20 (17)	47 (44)	76 (36)			
Obese	22 (18)	23 (21)	46 (22)			
**Marital status**				P = 0.735		
Single	1 (0.8)	1 (0.9)	3 (1.2)			
Married	109 (91.0)	100 (90.0)	194 (84.5)			
Divorced	2 (1.6)	1 (0.9)	1 (0.5)			
Widow/Widower	8 (6.7)	9 (8.1)	33 (14.3)			
**Education level**				P = 0.610		
Primary school	76 (63.0)	83 (75.0)	189 (82.0)			
High school	29 (24.1)	23 (21.0)	30 (13.0)			
Bachelor	10 (8.3)	3 (2.7)	10 (4.3)			
Master	4 (3.0)	1 (0.9)	2 (0.86)			
Doctorate	1 (0.83)	1 (1.9)	0 (0)			
**Smoking**				P = 0.297		
Current	21 (18)	18 (16)	54 (23.5)			
Former	24 (20)	17 (15)	45 (19.5)			
Never	74 (62)	76 (69)	132 (57)			
**Diabetes mellitus**				P < 0.001		
Yes	10 (8)	15 (13.5)	76 (33)			
No	110 (92)	96 (86.5)	155 (67)			
**Statin**				0.013		
Yes	0 (0)	38 (34)	112 (48.5)			
No	120 (100)	73 (66)	119 (51.5)			
**Aspirin**				< 0.001		
Yes	0 (0)	53 (47.8)	143 (62)			
No	12 (100)	58 (52.2)	88 (38)			
**Hypertension**				0.094		
Yes	12 (10)	22 (20)	40 (17)			
No	108 (90)	89 (80)	191 (83)			
**LDL-C, mg/d**L	120.83 ± 32.07	91.75 ± 40.07	98.03 ± 46.37	< 0.001	0.441	< 0.001
**HDL-C, mg/dL**	42.95 ± 8.61	46.39 ± 31.62	38.50 ± 17.10	0.417	0.006	0.158
**IFN γ, pg/mL**	0.38 (0.53)	0.00 (0.61)	0.41 (0.62)	0.113	0.249	0.765
**IL1α, pg/mL**	0.50 (0.19)	0.47 (0.66)	0.49 (0.62)	0.197	0.237	0.936
**IL1β, pg/mL**	0.56 (0.23)	0.59 (0.87)	0.57 (0.42)	0.985	0.575	0.437
**IL2, pg/mL**	2.70 (0.69)	2.51 (3.98)	2.79 (1.34)	0.974	0.644	0.465
**IL4 (pg/mL)**	1.81 (0.49)	2.16 (0.78)	2.02 (0.70)	0.045	0.970	0.029
**IL6, pg/mL**	1.04 (1.18)	1.25 (1.79)	1.51 (1.96)	0.148	0.525	0.003
**IL8, pg/mL**	4.49 (2.06)	4.89 (5.43)	5.72 (5.12)	0.116	0.760	0.245
**IL10, pg/mL**	0.78 (0.46)	0.75 (0.48)	0.71 (0.40)	0.847	0.017	0.002
**MCP1, pg/mL**	27.38 (32.46)	1.07 (92.38)	1.09 (77.80)	< 0.001	0.904	< 0.001
**TNF α, pg/mL**	1.78 (0.70)	1.67 (1.05)	1.79 (1.15)	0.197	0.839	0.321
**Depression score**	12.87 ± 10.01	10.68 ± 7.42	10.18 ± 7.70	0.076	0.58	0.007
**Depression**				1.00	0.049	0.033
Yes	37 (33)	36 (33)	52 (23)			
No	74 (67)	75 (67)	179 (77)			

^a^ Data are presented as mean ± SD, No. (%), or median (IQR).

**Table 2.** Cytokine Valuations in Depressed and Non-Depressed Subjects^a^

Variable, pg/mL, Median (IQR)	All Cases (n = 462)			Angiography-Positive and Negative (n = 342)		
	Depressed (n = 128)	Non-depressed (n = 328)	P Value	Depressed (n = 88)	Non-depressed (n = 254)	P Value
**INF γ**	0.44 (0.62)	0.35 (0.58)	0.223	0.44 (0.73)	0.41 (0.60)	0.379
**MCP1**	84.87 (96.95)	88.69 (84.17)	0.589	1.16 (86.35)	1.04 (82.29)	0.363
**IL1 α**	0.48 (0.60)	0.50 (0.63)	0.756	0.47 (0.60)	0.49 (0.64)	0.501
**IL1 β**	0.57 (0.32)	0.57 (0.39)	0.582	0.54 (0.60)	0.57 (0.46)	0.227
**IL2**	2.60 (1.19)	2.70 (1.16)	0.217	2.70 (3.78)	2.70 (1.47)	0.494
**IL4**	2.02 (.62)	1.98 (0.69)	0.529	2.10 (0.68)	2.09 (0.75)	0.869
**IL10**	0.75 (0.36)	0.72 (0.46)	0.338	0.73 (0.29)	0.72 (0.50)	0.566
**IL6**	1.62 (1.89)	1.27 (1.38)	0.031	1.60 (2.47)	1.41 (1.17)	0.169
**TNF α**	1.83 (0.92)	1.68 (0.95)	0.098	1.83 (1.19)	1.63 (1.10)	0.046
**IL8**	5.48 (6.75)	4.91 (3.14)	0.025	7.81 (9.80)	5.10 (3.90)	< 0.001

^a^ Abbreviations: IQR, interquartile range; INF γ, interferon γ; IL1α, interleukin-1α; IL1β, interleukin-1 β; IL2, interleukin-2; IL4, interleukin-4; IL6, interleukin 6; IL8, interleukin-8; IL10, interleukin-10; MCP-1, monocyte chemoattractant protein-1; TNF-α, Tumor necrosis factor.

**Table 3.** Adjusted Associations Between the Three Groups and Other Confounding Variables^a,b^

Dependent	Coefficient (β)	P Value
**IL4**		
Age	0.002	0.495
Sex	-0.089	0.173
Statin	-0.092	0.217
BMI	0.007	0.285
Healthy group	-0.311	0.000
Angio-negative group	-0.355	0.000
Angio-positive group	-	-
**IL6**		
Age	0.009	0.000
Sex	0.034	0.548
Statin	0.057	0.371
BMI	0.000	0.977
Healthy group	-0.270	0.000
Angio-negative group	-0.193	0.005
Angio-positive group	-	-
**IL10**		
Age	-00.002	0.992
Sex	0.057	0.191
Statin	0.024	0.629
BMI	-0.006	0.182
Healthy group	0.170	0.003
Angio-negative group	0.149	0.005
Angio-positive group	-	-
**MCP-1**		
Age	0.044	0.832
Sex	20.608	0.590
Statin	80.761	0.114
BMI	0.392	0.451
Healthy group	-880.571	0.832
Angio-negative group	-190.961	0.590
Angio-positive group	-	0.114

^a^ Abbreviations: BMI, body mass index; interlukin; MCP-1, monocyte chemoattractant protein-1.

^b^ P value for finding the regression model changes, when the variable was added. Coefficients (β) refer to how many SDs in cytokine (dependent variable) change in the model.

## 5. Discussion

Circulating markers of inflammation, such as CRP, serum amyloid A, IL-6, and IL-1 receptor antagonist, increase in CAD (12, 17). There appears to be a central role for inflammation in all phases of the atherosclerotic process (12, 16). Furthermore, some inflammatory cytokines such as IL-1 may stimulate production of other cytokines by central nervous system cells, including astrocytes and microglia, thereby, potentially promoting inflammation in the brain (4).

Cytokines released by immunological cells during viral infections or cancer therapy produce symptoms of sickness behavior, typically including decreased appetite, anorexia, fatigue and sleep disturbances, retardation of motor activity, reduced interest to physical and social environments, loss of libido, impaired cognitive abilities, dysphoria, anhedonia, and depressed mood (4).

Immune activation is associated with depression as well as increased numbers of circulating leucocytes and proinflammatory cytokines such as IL-1, IL-2 and IL-6 (6, 11). A hypothesis is that there is a positive feedback mechanism between depression and CAD. Increased concentrations of proinflammatory cytokines influence atherosclerotic plaque progression (12, 16) and sickness behavior. Sickness behavior can lead to inactive depressed life style that further exacerbates the risk of CAD.

After adjusting the effects of confounding factors with regression modeling among the three groups for IL-4, IL-6, IL-10, and MCP-1, a significant difference was found between the healthy and angiography-positive groups (Table 3), which may indicate that these biomarkers are associated with plaque inflammation (12, 31).

We found a significant difference between depressed and non-depressed subjects in serum levels of inflammatory cytokines and similarly we found that some of these cytokines were also significantly different between patients undergoing angiograms and healthy subjects.

Dowlati et al. undertook a meta-analysis of 16 studies for the role of cytokines in major depression and found that serum TNF-α and IL-6 were significantly higher in depressed versus non-depressed subjects (32). Our findings were in similar ranges.

Other studies have reported that serum IL-1β and TNF-α levels were not significantly higher in major depression disorders compared with normal subjects (33). We obtained the same result for TNF-α. Simon et al. have also reported a significant difference between depressed and non-depressed subjects in IL-8 (34).

IFN-γ, IL-6 and TNF-α increase the expression of indoleamine-2,3-dioxygenase (IDO) in immune-competent cells (35), an enzyme that can degrade tryptophan and may thereby lead to depressive symptoms (36). Our findings supported the association between elevations of proinflammatory cytokines such as IL-6 and TNF-α and major depression (4, 32). Moreover, proinflammatory cytokines were higher in CAD subjects, as previously reported (12, 16, 37).

One of the limitations in this study was the use of 50% cut-off threshold in defining a positive angiogram. Angiogram-negative subjects may progress to being angiogram-positive over time. Our observations were consistent with the hypothesis that there is a positive interaction between depressions.
